# Identification of the *Pol* Gene as a Species-Specific Diagnostic Marker for Qualitative and Quantitative PCR Detection of *Tricholoma matsutake*

**DOI:** 10.3390/molecules24030455

**Published:** 2019-01-28

**Authors:** Luying Shan, Dazhou Wang, Yinjiao Li, Shi Zheng, Wentao Xu, Ying Shang

**Affiliations:** 1 Yunnan Institute of Food Safety, Kunming University of Science and Technology, Yunnan 650500, China; shanluying1994@163.com (L.S.); wdz101545171@163.com (D.W.); 18487156903@163.com (Y.L.); zhengshi199201@163.com (S.Z.); 2 Beijing Laboratory of Food Quality and Safety, College of Food Science and Nutritional Engineering, China Agricultural University, Beijing 100083, China; xuwentao@cau.edu.cn

**Keywords:** *Tricholoma matsutake*, *Pol* gene, qualitative and quantitative PCR, DNA extraction

## Abstract

*Tricholoma matsutake* is a rare, precious, and wild edible fungus that could not be cultivated artificially until now. This situation has given way to the introduction of fake *T. matsutake* commodities to the mushroom market. Among the methods used to detect food adulteration, amplification of species-specific diagnostic marker is particularly important and accurate. In this study, the *Pol* gene is reported as a species-specific diagnostic marker to identify three *T. matsutake* varieties and 10 other types of edible mushrooms through qualitative and quantitative PCR. The PCR results did not reveal variations in the amplified region, and the detection limits of qualitative and quantitative PCR were found to be 8 ng and 32 pg, respectively. Southern blot showed that the Pol gene exists as a single copy in the *T. matsutake* genome. The method that produced the purest DNA of *T. matsutake* in this study was also determined, and the high-concentration salt precipitation method was confirmed to be the most suitable among the methods tested. The assay proposed in this work is applicable not only to the detection of raw materials but also to the examination of processed products containing *T. matsutake*.

## 1. Introduction

*Tricholoma matsutake* is an ectomycorrhizal agaricomycete predominantly associated with pines and oaks. It is a commercially valuable edible mushroom [1] with great significance, not only because of its delicate flavor but also because of its diverse biological properties [2], which include multiple immunostimulatory, hematopoietic, antineoplastic, antimutation, and antioxidation activities [3].

The growing number of *T. matsutake* consumers has steadily expanded market demands for this mushroom. On account of the continued deterioration of ecological systems and the environment and excessive picking, the natural productivity of this gourmet mushroom has gradually declined. To date, no artificial cultivation method has yet been developed for *T. matsutake* due to the lack of information concerning precise soil requirements and cues for sporophore formation [4]. Therefore, wild *T. matsutake* and its products are in a short supply in the market, and, as such, its economic value has risen sharply. To address demands for the mushroom and reap higher profits, merchants frequently pass off counterfeit or adulterated *T. matsutake* products as genuine items, for example, sliced dried *Agaricus blazei* [5]. Establishing an effective and convenient method for identifying authentic *T. matsutake* is an urgent necessity.

Traditional methods of identifying *T. matsutake* are generally divided into two categories, namely, morphological [6] and physicochemical methods [7], and each category presents some limitations [8]. Morphological methods are effective in identifying fresh and integrated sporophores. Unfortunately, identification becomes much more difficult when applied to processed products on account of the destruction of the morphological characteristics of the mushroom. Physicochemical methods, such as gas chromatography, high-performance liquid chromatography, and mass spectrometry, require selection of a unique compound or fingerprint to represent *T. matsutake*, and the detection result is easily influenced by variety, habitat, and processing method [9,10]. Thus, two or more physicochemical means are commonly combined to obtain a valid result. If an isomeride exists in the analyte, detection becomes even more complicated. When the unique compound is added artificially, the physicochemical method may fail.

Rapid developments in modern biotechnology have enabled the wide use of molecular biological methods for authentication due to their accuracy, convenience, and speed. PCR-based methods, in particular, have been broadly adopted by many laboratories and fields [11,12]. Compared with conventional PCR, quantitative PCR technology has realized the leap of PCR from qualitative to quantitative, and it has higher specificity, effective resolution and higher degree of automation, and widely used in many fields such as gene expression research, transgenic research, drug efficacy assessment, pathogen detection and food composition analysis, especially. Therefore, real-time quantitative PCR detection is considered to be an easy-to-use, accurate, specific, sensitive, and quantitative method [13,14,15]. Furthermore, the species-specific diagnostic marker (also denoted as endogenous reference gene in some literatures) is a significant parameter during PCR amplification, which can evaluate the quality of the extracted DNA and provide the means to quantify the amount of the tested DNA substance in the processed food samples [15]. 

Identification of species-specific diagnostic marker, which requires species specificity, a consistent and low copy number, and low heterogeneity in the same species [13,14], has principally focused on crops. Numerous species-specific diagnostic marker of have been developed and reported [13], *vicK* for *Staphylococcus aureus* [15], Ribosomal Protein *L21* [16] for genetically modified (GM) wheat; *CruA* [17], *PEP* [18], *HMG-I/Y* [19], *FatA* [20], and *BnAccg8* [21] for canola; *lectin* [17,22], *β-actin* [17], and *hsp* [23] for soybean; *Cotton-ppi-PPF* [24], *ACP*1 [25], *Sad1* [26], and *SAH7* [27] for cotton; *hmga* [28,29,30], *10kDa zein* [30,31,32,33], *Ivr1* [23,30,34,35], *zSSIIb* [22,36,37], and *Adh*l [30] for maize; and *gos9* [38], *RBE4* [39], and *SPS* [40] for rice. Appropriate reference genes under abiotic stress of annual ryegrass also was selected [41]. However, species-specific diagnostic markers for *T. matsutake* have yet to be established or reported.

Extracting high-quality DNA from *T. matsutake* fruiting bodies is crucial for downstream molecular experiments. At present, the cetyltrimethyl ammonium bromide (CTAB) method, which is especially suitable for plant DNA extraction, is commonly used to extract mushroom DNA [42]. However, high-quality DNA from *T. matsutake* fruiting bodies is difficult to acquire using this method because the mushroom contains thick cytoderms or capsules [43] and is rich in polyphenols and viscous polysaccharides. Thus, exploring a suitable DNA isolation method for *T. matsutake* fruiting bodies is an important endeavor.

In this work, the *Pol* gene, which encodes RNase H and integrase, was selected as a candidate species-specific diagnostic DNA marker for *T. matsutake* through sequence alignment and BLAST. The gene was verified to be a valid species-specific diagnostic marker given its features of appearance as a single copy in genomic DNA, good species specificity, and real-time quantitative detection limit as low as 32 pg. A suitable method for extracting the DNA of *T. matsutake*, i.e., high-concentration salt precipitation method, was also determined. The benefits of the present study are multifold: it provides a convenient and accurate approach for detecting *T. matsutake* and its related processed products, it aids efforts to improve food safety and detect food adulteration, and it develops an effective technique for detecting adulterations in the wild-mushroom market.

## 2. Results and Discussion

### 2.1. Comparison of DNA Extraction Methods

**Figure 1:**
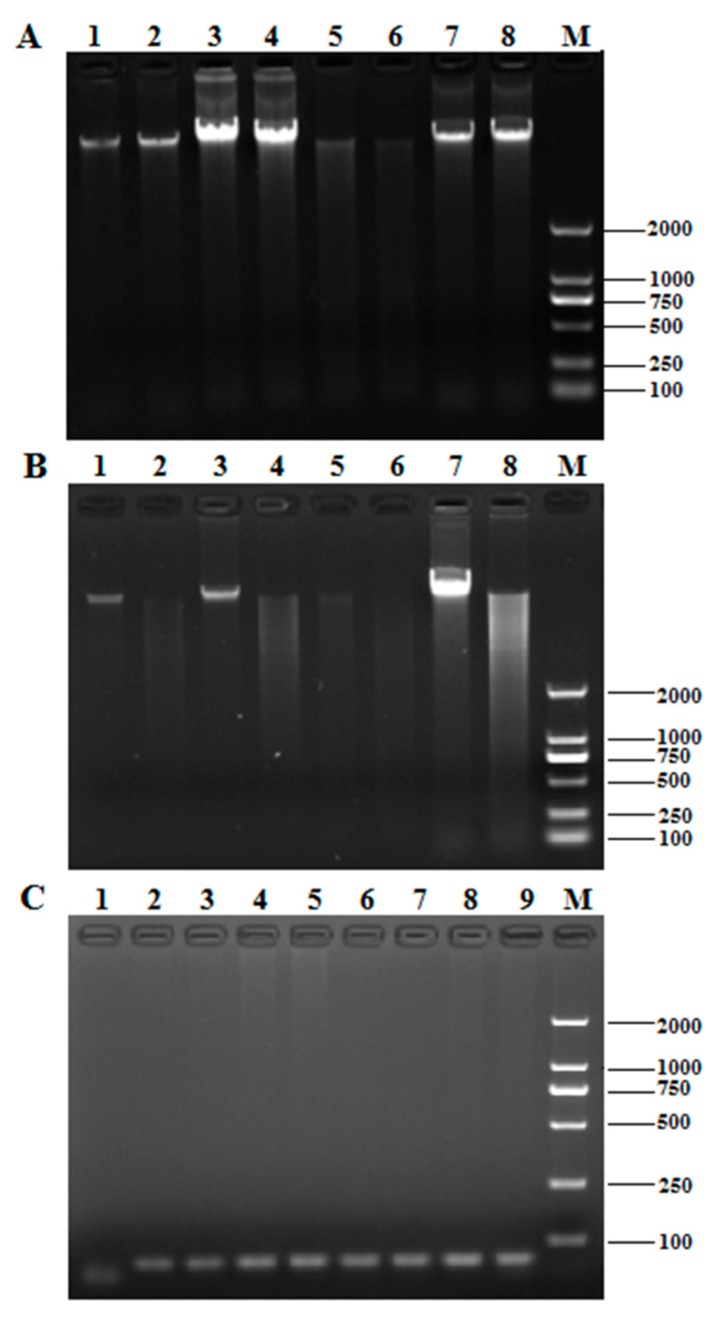
The effect of different DNA extraction methods. (**A**) The electrophoresis profile of *T. matsutake* genomic DNA; (**B**) the digestion result of *T. matsutake* genomic DNA. 1–2: cetyltrimethyl ammonium bromide (CTAB); 3–4: SDS-CTAB; 5–6: high-salt enzymolysis; 7–8: fungal genomic DNA isolation kit; M: DNA marker DL 2000; (**C**) the electrophoresis profile of PCR products of Tricholoma matsutake genomic DNA amplified by 18S universal primers. 1: negative control; 2–3: CTAB; 4–5: SDS-CTAB; 6–7: high-salt enzymolysis; 8-9: fungal genomic DNA isolation kit; M: DNA marker DL 2000.

The DNA of *T. matsutake* fruiting bodies from Yunnan was obtained using the CTAB, SDS-CTAB, high-concentration salt precipitation, and kit methods. The results of 1% agarose gel for genomic DNA (Figure 1A) indicated that the four DNA extraction methods could successfully extract genomic DNA with good integrity. All of the OD260/280 and DNA concentrations obtained are listed in Table 1, there are significant differences in the DNA concentrations, and the OD260/280 ratio of the High-concentration salt precipitation method compared with other three extraction methods reveals that the purity of genomic DNA extracted by this method is the highest.

The restriction enzyme digestion result in Figure 1B showed that the genomic DNA extracted by the four methods could be digested by *EcoR* I, and the products were in a dispersive state. However, the digestibility of genomic DNA extracted by the CTAB, SDS-CTAB, and kit methods was lower than that of the high-concentration salt precipitation method, and there were a large number of large fragments, which showed brighter digestion products in the upstream part of the lanes, while the high-concentration salt precipitation method was more uniform. It revealed the high-concentration salt precipitation method was superior in quality and has less enzyme inhibitors.

The PCR products of the 18S rRNA region amplified with the fungal universal 18S primers were staining to identify whether the extracted *T. matsutake* genomic DNA can be used for subsequent PCR amplification. As shown in Figure 1C, the 18S rRNA fragments were efficiently amplified using the DNA extracted by the four methods. 

In summary, by integrating the above four indicators shown in Table 1 and Figure 1, all genomic DNA extracted by the four methods could meet with the requirements of the PCR experiment, however, the high-concentration salt precipitation method was confirmed to be the most suitable DNA extraction way among the methods tested.

### 2.2. Species-Specific Diagnostic Marker of *T. Matsutake*

Among the genes selected, the *Pol* gene (*Tricholoma matsutake pol* gene for polyprotein encoding RNase H and integrase, Genbank No. AB016926) showed no homology with other genes from different varieties of the mushroom.

It has been reported that retrotransposons have been incorporated into the genome of their hosts and inherited to the host progenies since the earliest establishment of their parasitism. In a previous study, this *Pol* gene was a retroelement from *T. matsutake*, which related to RNase H and integrase of retrotransposons [44]. The reverse transcriptase domain was found in *T. matsutake* worldwide, this finding suggested that retroelements associate with ectomycorrhizal basidiomycetes and might be useful as genetic markers for identification, phylogenetic analysis, and mutagenesis of this fungal group [45]. After the BLAST analysis of *Pol* gene, it just confirmed the statement above. Hence, we chose the *Pol* gene as the candidate species-specific diagnostic marker of *T. matsutake*.

### 2.3. Species Specificity of Qualitative PCR aSSAYS

**Figure 2:**
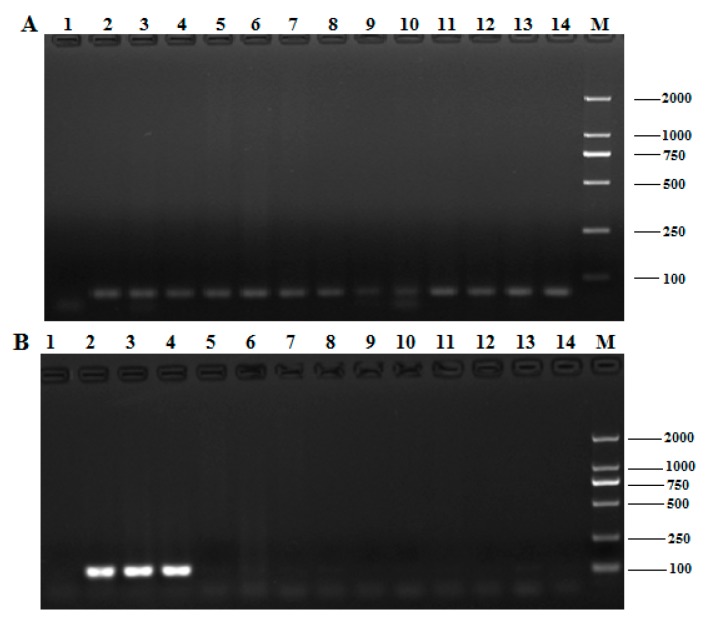
Specificity of the *Pol* gene detection in qualitative PCR. (**A**) The electrophoresis profiles of the DNA products amplified by the 18S rDNA; (**B**) the species specific identification of the *Pol* gene from *T. matsutake* by conventional PCR. 1: negative control, 2: Yunnan Shangri-La *T. matsutake*, 3: Sichuan Ganzi *T. matsutake*, 4: Jilin Yanbian *T. matsutake*, 5: *Russula virescens*, 6: *Agaricus deliciosus*, 7: *Boletus speciosus Forst*, 8: *Termitornyces albuminosus*, 9: *Agaricus blazei*, 10: *Agrocybe aegerita*, 11: *Lentinula edodes*, 12: *Pleurotus eryngii*, 13: *Flammulina velutipes*, 14: *Pleurotus ostreatus*, M: DNA marker DL 2000.

The genomic DNA isolated from 10 non-*T. matsutake* species (*R. virescens*, *A. deliciosus*, *B. speciosus*, *T. albuminosus*, *A. blazei*, *L. edodes*, *P. eryngii*, *F. velutipes*, and *P. ostreatus*) and three *T. matsutake* strains (Yunnan, Sichuan, and Jilin) was subjected to 18s rDNA amplification (Figure 2A). The 18s rDNA primer pair was used to confirm all the extracted DNA can be effectively amplified, and the quality of the extracted DNA clearly met the conditions of PCR (Figure 2A). 

The primer pair *Pol*-F/R was applied to the qualitative PCR of the *Pol* gene; PCRs were also conducted using the genomic DNA of the 13 mushroom samples indicated above. Electrophoretic analysis of all qualitative PCR products (Figure 2B) revealed no objective product, except in the PCR products of the three *T. matsutake* samples. The results confirm that the qualitative PCR applied in this work are highly specific for *T. matsutake*.

### 2.4. Homology Analysis of the Pol Gene among Different *T. Matsutake* Varieties

**Figure 3:**
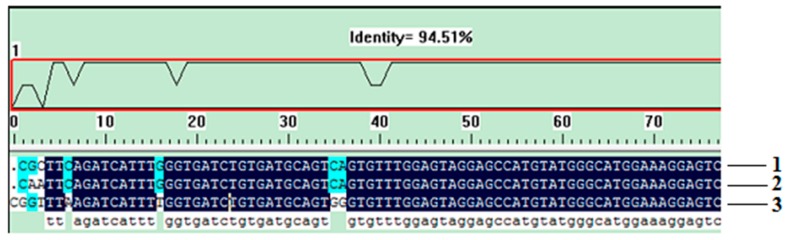
The sequencing result of *Pol* gene in different samples. 1, Yunnan Shangri-La *T. matsutake*; 2, Sichuan Ganzi *T. matsutake*; 3, Jilin Yanbian *T. matsutake*.

An excellent species-specific diagnostic marker should have low heterogeneity and a consistent copy number in the same species. We carried out qualitative PCR using identical amounts of DNA from the three *T. matsutake* strains to determine whether the *Pol* gene undergoes any sort of variation. Amplification products with identical sizes and relative intensities were obtained for all varieties after qualitative PCR (Figure 2B, lines 2–4), and the slight differences, which were attributed to the quality of the isolated DNA [13], were considered negligible. As shown in Figure 3, the homologous similarity sequence identity between the PCR products and the reference *Pol* gene was 94.51%. These results indicate that the *Pol* gene did not show the sequence variation among the *T. matsutake* varieties studied.

### 2.5. Confirmation of the Pol Gene Copy Number by Southern Blot 

**Figure 4:**
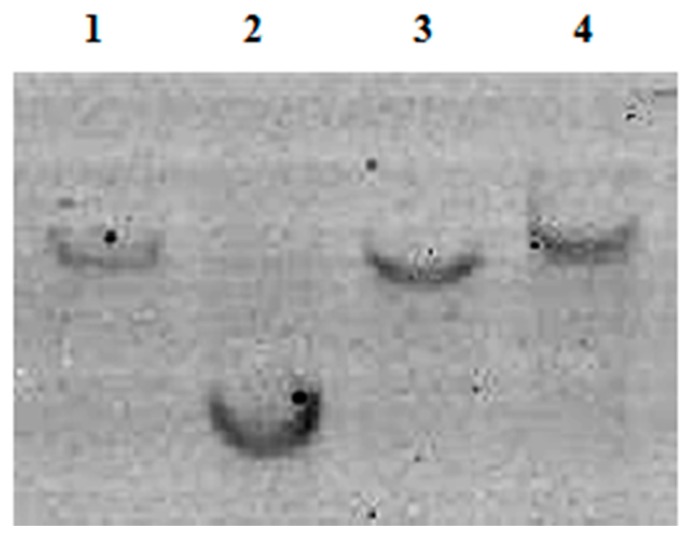
Southern blot result. 1, Jilin Yanbian *T. matsutake* digested by EcoR I; 2, Jilin Yanbian *T. matsutake* digested by Not I; 3, Yunnan Shangri-La *T. matsutake* digested by EcoR I; 4, Yunnan Shangri-La *T. matsutake* digested by Not I.

Besides species specificity, low sequence variation, and a consistent copy number, an excellent species-specific diagnostic marker is expected to possess a low copy number. Therefore, using Southern blot, we analyzed the copy number of the *Pol* gene in two *T. matsutake* varieties gathered from Yunnan and Jilin Provinces. Whether *Hind* III or *Eco*R I was used to digest the genomic DNA of *T. matsutake*, only one hybridization band was found in the nylon membrane (Figure 4), which means the *Pol* gene is only present as a single copy in the *T. matsutake* genome.

### 2.6. Sensitivity of the Qualitative and Taqman-Based Real-Time Quantitative PCR Assays

**Figure 5:**
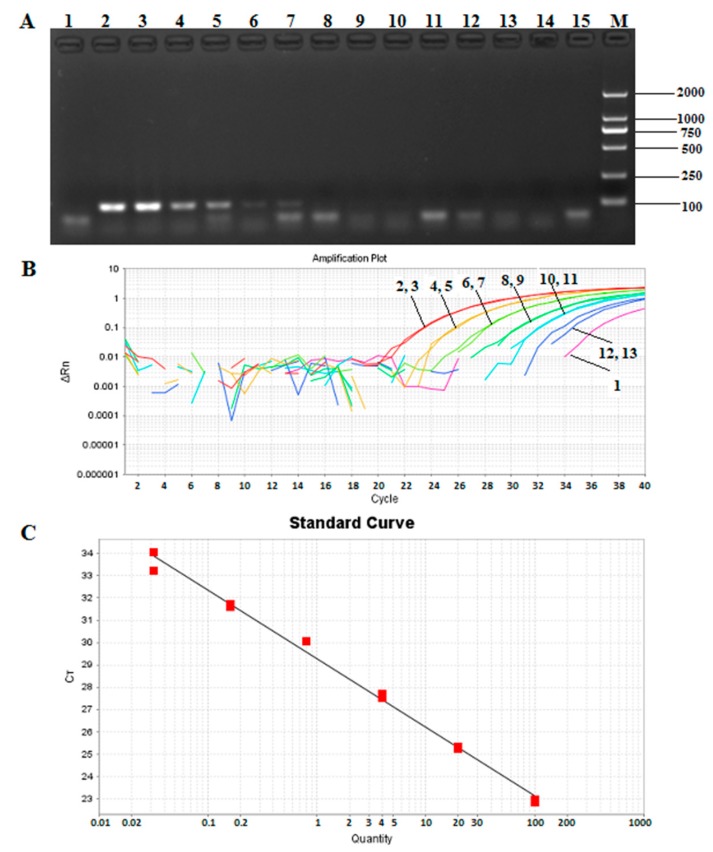
Sensitivity of the *Pol* gene detection in qualitative and Taqman quantitative PCR. (**A**) Sensitivity detection of *Pol* gene by conventional PCR. 1: negative control; 2–3: 200 ng; 4–5: 40 ng; 6–7: 8 ng; 8–9: 1.6 ng; 10–11: 0.32 ng; 12–13: 0.064 ng; 14–15: 0.0128 ng; M: DNA marker DL 2000; (**B**) the amplification curves of *Pol* gene by quantitative PCR. 1: negative control; 2–3: 100 ng; 4–5: 20 ng; 6–7: 4 ng; 8–9: 0.8 ng; 10–11: 160 pg; 12–13: 32 pg; (**C**) the standard curve of the sensitivity detection of *Po*l gene by qPCR.

Genomic DNA from *T. matsutake* was diluted five times from 200 ng/µL to 12.8 pg/μL over a gradient, and the results (Figure 5A) showed a detection limit of 8 ng for qualitative PCR. With the same way, in Figure 5B, the sensitivity of Taqman quantitative PCR was found to be 32 pg. A standard curve of the *Pol* gene was then generated by using the proposed quantitative PCR system, and a linear relationship (*R*^2^ = 0.993) with a slope of −3.081 was determined between the DNA quantities and Ct values (Figure 5C). 

### 2.7. Application of the Pol gene to Detect Processed *T. Matsutake* Products

**Figure 6:**
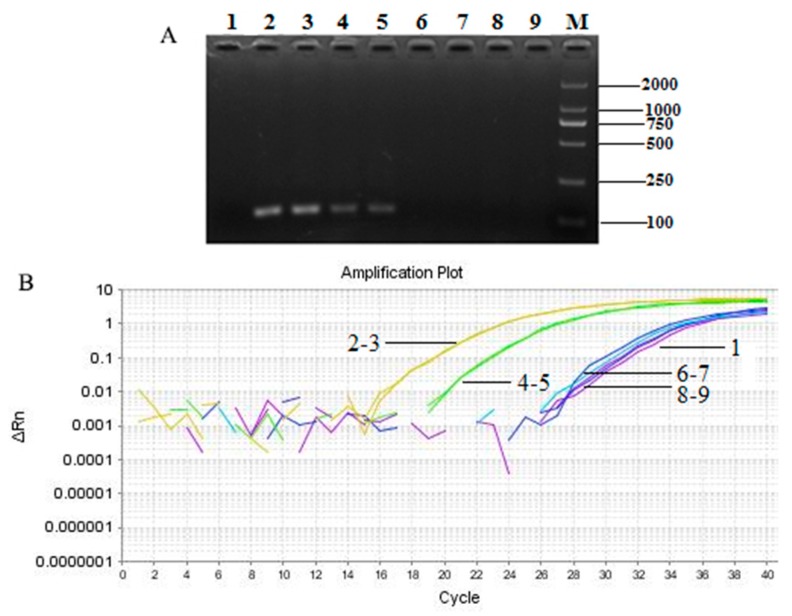
The detection of *T. matsutake* component in different kinds of food samples by qualitative (**A**) and real-time quantitative (**B**) PCR. 1: negative control; 2–3: positive control (Yunnan Shangri-La *T. matsutake*); 4–5: *T. matsutake* biscuits; 6–7: *T. matsutake* sauce; 8–9: Oil field *T. matsutake*; M: DNA marker DL 2000.

We used the established Taqman-based PCR system to detect the source of *T. matsutake* in processed products, including mushroom biscuit, oil, and sauce. The target products could be amplified by employing Taqman-based quantitative PCR with the primer *Pol*-F/R and the probe *Pol*-P. The PCR results (Figure 6A) were consistent with the ingredients listed on the packaging of each products. The results of real-time quantitative PCR (Figure 6B) were in keeping with those of qualitative PCR. So, the results of both assays were prove that *T. matsutake* biscuits tested contained *T. matsutake* and the *Pol* gene was a practical and precise species-specific diagnostic marker for *T. matsutake* in highly processed foods, and affirmed its absence, as necessary.

## 3. Materials and Methods

### 3.1. Materials

The following mushroom species were purchased from local farmers’ markets: *Russula virescens*, *Agaricus deliciosus*, *Boletus speciosus*, *Termitornyces albuminosus*, *Agaricus blazei*, *Agrocybe cylindracea*, *Lentinula edodes*, *Pleurotus eryngii*, *Flammulina velutipes*, and *Pleurotus ostreatus*. Three *T. matsutake* varieties strains collected from the provinces of Yunnan, Sichuan, and Jilin, China, were kindly supplied by all China Federation of Supply and Marketing Cooperative’s Kunming Institute of Edible Fungi. All of the samples were collected in the quantity of 200 g.

Three processed products labeled containing *T. matsutake* component, including mushroom biscuit, oil, and sauce, were gathered from Internet and local markets in Kunming, Yunnan province. They were used to verify the application of this selected species-specific diagnostic marker.

### 3.2. Genomic DNA Extraction

Four methods, including an improved CTAB method [46], the SDS-CTAB method [47], high-concentration salt precipitation method [48], and the kit method (Rapid Fungi Genomic DNA Isolation Kit, B518229, Sangon, Shanghai, China), were compared to determine the most suitable protocol for extracting the genomic DNA of *T. matsutake*. The *T. matsutake* fruiting bodies from Yunnan was used as the sample, each method was performed in triplicate. First, added the equal silica into 5 g mushroom sample, and then grounded them with liquid nitrogen. The target genomic DNA was isolated from the 0.2 g sample powder. All DNA extraction steps were performed in accordance with the references and manufacturer protocol. At last, the DNA was eluted in 50 μL EB buffer.

The subsequent DNA extraction was followed the selected optimal method. All of the OD260/280 and DNA concentrations were measured with a NanoDrop2000 spectrophotometer (Thermo Scientific, Waltham, MA, USA), the quality of genomic DNA was further analyzed on 1% agarose gel with ethidium bromide (0.1 μg /mL), and run with 1 × TAE buffer. Finally, the DNA solutions were stored at −20 °C.

### 3.3. Enzyme Digestion of *T. Matsutake* Genomic DNA

The DNA digestion was performed in a 20 μL reaction system containing 2 μL of 10× H Buffer, 1 μL *EcoR* I (15 U/μL) (TaKaRa Biotechnology Co. Ltd., Dalian, China), 3 μg genomic DNA, and added ultrapure water to 20 μL, and put the tube in a water bath (37 °C) for 2 h, after that added 2 μL 10× loading Buffer (Tiangen, Beijing, China) to stop the reaction. The digestion products was further analyzed on 1% agarose gel with ethidium bromide (0.1 μg /mL), and run with 1× TAE buffer.

### 3.4. Southern Blot

Complete enzyme cleavage of DNA from *T. matsutake* fruiting bodies obtained from Yunnan and Jilin was performed using *Hind* III and *Eco*R I, respectively, according to the manufacturer’s instructions (TaKaRa) to ascertain the copy number of the *Pol* gene in the same species. The cleaved DNA was separated by 0.8% garose gel in 1×TAE at a constant voltage of 20 V overnight. Thereafter, the DNA fragments were transferred to a nylon membrane (Amersham Biosciences Shanghai Ltd., Darmstadt, Germany) from the 0.8% agarose gel. A 775 bp DNA fragment of the *Pol* gene was amplified with *Southern blot*-F/R and used as the hybridized probe, which was labeled with DIG-dUTP (DIG Hybridization Detection Kit, Mylab Co., Beijing, China). Pre-hybridization was carried out at 42 °C for 2 h, after which the pre-hybridization solution was poured off. The probe was denatured into single-strand DNA for 10 min at 100 °C and then cooled for 5 min. Exactly 4 µL of the denatured probe and 8 mL of the hybridization solution were added to a hybridization bag, and hybridization was performed at 42 °C overnight. Finally, the nylon membrane was washed twice with 3× SSC/0.5% SDS, and autoradiography was performed for 2–3 days.

### 3.5. Species-Specific Diagnostic Marker Selection of *T. Matsutake*

Genes belonging to *T. matsutake* were sought in Genbank, and several genes were preliminarily selected as detection targets according to their detailed gene information. Afterword, BLAST analysis was performed to determine the homology of the targets. The gene that with the lowest homology compared with all the DNA sequences in Genbank was selected as the candidate.

### 3.6. Primers and Probe

All primers and TaqMan fluorescent dye-labeled probes were designed using ABI Prism Primer Express version 3.0 software (Applied Biosystems, Foster City, CA, USA) and synthesized by Sangon Co. Ltd. (Shanghai, China).

The universal primer 18S rDNA-F/R was used to evaluate DNA quality. The *Pol* gene was assessed through qualitative PCR with the primer *pol*-F/R and through quantitative PCR with the primer *pol*-F/R, as well as the probe *pol*-P. Southern blot-F/R was used for Southern blot assay. The detailed sequences of these primers and probe are listed in Table 2.

### 3.7. PCR Conditions

Qualitative PCR was performed using an ABI SimpliAmp thermal cycler (Applied Biosystems) in a 25 µL reaction system containing 2.5 μL of 10×buffer, 0.2 mM dNTP, 0.4 μM of each primer, 2.5 units of Taq DNA Polymerase (TaKaRa Biotechnology Co. Ltd.), 100 ng DNA template, and 16.3 µL of ultrapure water. The 18S rDNA amplification was carried out to test DNA quality via the following program: 5 min at 95 °C; 40 cycles of 30 s at 95 °C, 30 s at 58 °C, and 30 s at 72 °C; and 10 min at 72 °C. During *Pol* gene amplification, the procedures applied for 18S rDNA amplification were followed, but annealing was optimized at 56 °C, 58 °C, 60 °C, 62 °C, and 64 °C separately. After comparison, the annealing temperature 60 °C was chosen. 

The PCR amplified products were analyzed on 2% agarose gel with ethidium bromide (0.1 μg/mL), and run with 1× TAE buffer. The PCR products, that conducted the homology analysis, were sequenced using *pol*-F/R separately by Sangon (Shanghai, China) company.

Real-time quantitative PCR of the *Pol* gene was also conducted in an ABI StepOne Plus Real-Time System (Applied Biosystems, Thermo Fisher Scientific, Waltham, MA, USA) using the TaqMan probe methods. TaqMan probe-based qPCR was performed using a 20 µL reaction system containing 1×TaqMan Gene Expression Master Mix (TaKaRa, Dalian, China), 200 nM primers, 200 nM probe, and 50 ng of DNA. Each sample was quantified twice for each biological replicate.

### 3.8. Sensitivity of the Qualitative and Taqman-Based Real-Time Quantitative PCR Assays

Genomic DNA from *T. matsutake* was diluted seven times from 200 ng/µL to 12.8 pg/μL (five-fold serial dilutions) over a gradient using the non-*T. matsutake* DNA, and a series of PCRs with 1 µL DNA sample were conducted to determine the detection limit of qualitative PCR, and the products were analyzed using 2% agarose.

To evaluate the sensitivity of Taqman-based quantitative PCR, genomic DNA was serially diluted six times to final concentrations ranging from 100 ng/μL to 32 pg/μL (five-fold serial dilutions) using the non-*T. matsutake* DNA, and with 1 µL DNA sample, the detection limit of Taqman quantitative PCR was determined.

## 4. Conclusions

In conclusion, this work demonstrated that High-quality DNA from *T. matsutake* fruiting bodies was obtained following the high-concentration salt precipitation method, and the *Pol* gene was selected and validated as an ideal species-specific diagnostic marker for the PCR-based detection of *T. matsutake* sources after assay of its species specificity, copy number, high homology in different varieties, and sensitivity. The detection limit of Taqman-based quantitative PCR analysis was 32 pg, which means this method could be used to detect processed *T. matsutake* products containing low amounts of the target genomic DNA.

## Figures and Tables

**Table 1:** Comparison of purity and concentration of DNA extracted by four methods ($\overset{–}{a}$ ± SD, *n* = 3).

Method	OD260/280	Concentration (ng/μL)
CTAB	2.01 ± 0.02 ^a^	983.33 ± 2.89 ^a^
SDS-CTAB	1.97 ± 0.01 ^a^	1138.33 ± 2.89 ^b^
High-concentration salt precipitation	1.88 ± 0.02 ^b^	676.67 ± 0.01 ^c^
Kit method	1.66 ± 0.11 ^c^	163.33 ± 2.89 ^d^

^a–d^ the upper letter of the OD260/280 and DNA concentrations indicates a significant difference (Duncan test, *p* < 0.05).

**Table 2:** Primers used in qualitative and quantitative PCR.

Primer Name	Primer Sequence (5′→3′)	Length	Product Size (bp)	Reference
18S-F	CCTGAGAAACGGCTACCAT	19	80	This study
18S-R	ATCTTCACTACCTCCCCATTCTG	23		
*pol*-F	GACTCCCATACTGAAGCCAAT	21	107 (from 69 to 175 bp)	
*pol*-R	ACTCCTTTCCATGCCCATAC	20		
*pol*-probe	(FAM)-TGGCTCCTACTCCAAACACTGACAC-(TAMRA)			
*Southern blot*-F	CGTGATGGATGGAATACCTGT	21	775 (from 563 to 1157 bp)	
*Southern blot*-R	GTGTACCCCCCCTTAGACTGA	21		
